# The value of sST2 in risk stratification and short-term prognosis of acute pulmonary embolism: a pilot study focusing on intermediate-risk subgroups

**DOI:** 10.3389/fcvm.2025.1588996

**Published:** 2025-07-02

**Authors:** Jian Wang, Zhen Liu, Yan Jiao, Yanli Cheng, Jinlong Li

**Affiliations:** Department of Cardiology, Binzhou Medical University Hospital, Binzhou, China

**Keywords:** soluble growth stimulation expressed gene 2 (sST2), risk stratification, acute pulmonary embolism, intermediate-risk, prognosis

## Abstract

**Background:**

Intermediate-risk acute pulmonary embolism (APE) represents a heterogeneous group that is temporarily hemodynamically stable and still has a high mortality. The aim of this study was to assess the predictive value of soluble growth stimulation expressed gene 2 (sST2) in risk stratification and short-term prognosis in this group.

**Methods:**

This retrospective observational study included 128 patients with intermediate-risk APE between February 2020 to November 2023. Univariate or multivariate analysis were carried out for exploring the associations of sST2 with risk stratification and adverse event. Univariate logistic regression analysis and characteristic curve (ROC) were performed.

**Results:**

Compared with the intermediate-low risk group, higher sST2 level (25.8 ng/ml vs. 11.5 ng/ml, *P* < 0.001) and more adverse events (28.2% vs. 8%, *P* = 0.006) were observed in the intermediate-high risk group. Univariate logistic regression analysis showed that sST2 was associated with higher risk stratification (OR = 1.085, 95%CI 1.042–1.129, *P* < 0.001) and adverse events (OR = 1.049, 95%CI 1.027–1.072, *P* < 0.001). For intermediate-high risk stratification prediction, the AUC (area under the curve) was 0.754 (95% CI: 0.671–0.837, *P* < 0.001) using sST2 and the optimal probability of cut-off value was 16.20 ng/ml. For adverse events prediction, the AUC was 0.832 (95% CI 0.751–0.913; *P* < 0.001), while the optimal cut-off value was 16.20 ng/ml.

**Conclusions:**

sST2 is associated with risk stratification and poor short-term prognosis for intermediate-risk APE, and it is a promising new biomarker that may contribute to further stratification for intermediate-risk subgroups and identification of individuals with a propensity to develop adverse events during hospitalization.

## Introduction

Acute pulmonary embolism (APE) is a disease of pulmonary circulation disorder caused by obstruction of the trunk or branches of the pulmonary artery which can range from mild disease with no symptoms, to hemodynamic instability and mortality (1). Pulmonary embolism (PE) may cause 300,000 deaths per year in the United States, more than 370,000 deaths in six European countries (2) and even in China, APE is much more common than it was 10 years ago (3). Therefore, early recognition, early diagnosis and timely treatment of APE are essential. Risk stratification of patients with APE is mandatory for determining the appropriate therapy. High-risk APE is defined as hemodynamic instability, and thrombolytic therapy can significantly reduce mortality. For low-risk patients, parenteral or oral anticoagulation is an adequate treatment with a lower mortality rate (1%) (4). Nevertheless, intermediate-risk patients have a very heterogeneous prognosis. Among intermediate-risk patients, it is crucial to identify the subgroup of patients at increased risk of early death who may benefit from early reperfusion therapy. In the intermediate-risk group, indicators of risk include pulmonary embolism severity index (PESI) or simplified pulmonary embolism severity index (sPESI), right ventricular dysfunction (RVD), and laboratory elevated cardiac troponin or N-terminal pro-brain natriuretic peptide (*N*T-proBNP). And the latter are susceptible to many factors, leading to inaccuracy of risk stratification (5).

Soluble growth stimulation expressed gene 2 (sST2) is a member of the interleukin receptor family and participates in a broad range of biological processes relevant to cardiovascular disease (6). Compared with the commonly used markers brain natriuretic peptide (BNP), NT-proBNP, and troponin, sST2 was not significantly correlated with multiple factors such as age, gender, renal function and body mass index. Martinez-Rumayor et al. (7) reported that concentrations of sST2 were elevated in many patients with pulmonary diseases. In adults, circulating sST2 is associated with higher pulmonary artery pressure and RVD (8). Therefore, APE and its risk stratification may be associated with sST2, which has been confirmed by recent studies (9). However, the extent of sST2 elevation in intermediate-risk subgroup patients and its association with risk stratification and short-term prognosis remain unclear. The aim of this study was to assess the predictive value of sST2 in risk stratification and short-term prognosis in this group.

## Materials and methods

### Study population

From February 2020 to November 2023, 128 consecutive patients with a diagnosis of APE and no more than 14 days of symptom onset were enrolled in Binzhou Medical University Hospital. The inclusion criteria: (1) APE confirmed by computed tomography pulmonary angiography (CTPA). (2) Patients in the intermediate-risk group who meet the 2019 ESC Guidelines for the diagnosis and management of acute pulmonary embolism; the intermediate-high risk group was defined as patients with hemodynamic stability and sPESI ≥1, presenting with both RVD and elevated laboratory biomarkers, whereas patients exhibiting only one of these features (either RVD or elevated laboratory biomarkers) are classified into the intermediate-low risk group. Exclusion criteria included: (1) recurrent pulmonary embolism. (2) severe hepatic and renal insufficiency; (3) comorbid severe infection, acute myocardial infarction, heart failure, chronic obstructive pulmonary disease, acute ischemic cerebral infarction, tumor, blood diseases and immune diseases; (4) incomplete clinical data. Baseline characteristics as well as the patients’ treatment and in-hospital course were confirmed by the study physicians.

All patients were divided into 2 subgroups: intermediate-high risk (*N* = 78) and intermediate-low risk (*N* = 50) according to risk stratification; adverse event group (*N* = 26) and no adverse event group (*N* = 102) according to whether adverse events occurred in the hospital. An adverse event is defined as an in-hospital death or one of the following: the need for thrombolytic therapy, catecholamines to maintain blood pressure, mechanical ventilation, interventional therapy, cardiopulmonary resuscitation.

This study was conducted in compliance with the Declaration of Helsinki principles. The study was approved by the Ethics Committee of Binzhou Medical University Hospital (2023-LW-114). The participants provided their written informed consent to participate in this study. Besides, all treatment decisions were made prior to our evaluation.

### Biomarker testing

Venous plasma and serum samples were collected from all participants on admission. Plasma sST2 levels were measured by time resolved fluorescent immunochromatographic assay. The sST2 test was performed after the diagnosis of pulmonary embolism had been made (all within 24 h of admission to the hospital). CTnI and NT-proBNP levels were measured by fluorescent magnetic particle enzyme immunoassay and quantitative electrochemiluminescence assay respectively. The investigator responsible for the measurements was not aware of the patient's baseline parameters or clinical course. CTnI was defined as elevated if it was greater than 0.4 ng/ml. For NT-proBNP, the threshold value for elevation is 600 pg/ml.

### Diagnostic criteria for RVD

Imaging evidence included echocardiography or CTPA suggestive of RVD, with echocardiography consistent with the following findings: (1) right ventricular dilatation (right ventricular end-diastolic internal diameter/left ventricular end-diastolic internal diameter ≥1.0); (2) reduced right ventricular free wall motion; (3) increased tricuspid regurgitation; and (4) decreased tricuspid annular plane systolic excursion (<16 mm). CTPA examination was consistent with the following: right ventricular dilatation (right ventricular end-diastolic internal diameter/left ventricular end-diastolic internal diameter ≥1.0) found at the level of the four-chamber heart.

### Statistical analysis

Continuous variables with normal distribution were expressed as mean ± SD and median with P25–P75 was used to express the anormal distribution data. Student's t-test or non-parametric test was used for the comparison between groups. Categorical data were represented by numbers (%) and *χ*^2^ test was used for comparisons between groups. Univariate analysis was carried out for exploring the associations of sST2 with risk stratification and prognosis considering demographic and clinical information. In multivariate logistic regression, gender, age, and variables that were significant in univariate analysis were included for adjusting. ROC curves were drawn for the screening performance characteristics of sST2 in intermediate-high risk and poor prognosis. *P* < 0.05 was considered significant. Statistical analysis was performed with IBM SPSS27.0.

## Results

Among 128 patients included in the study, the average age was 66.6 ± 9.0 years, and 42.2% were male. More than half of patients (60.9%, 78/128, 95% CI 52.4%–69.5%) were classified in the intermediate-high risk group. A total of 26 patients (20.3%, 26/128, 95% CI 13.2%–27.4%) developed adverse events (as defined in the methods), of which 22 occurred in the intermediate-high risk group and 4 in the intermediate-low risk group. sST2 levels were 16.0(10.1, 32.1) ng/ml, and 27 patients (21.1%) had abnormally elevated (35 ng/ml). Level of NT-proBNP was 1412.0 (825.0, 3,772.3) pg/ml. The percentage of occurrence of dyspnea was high (93.8%). Disease histories showed that the population were with low prevalence of diabetes, tumor and cerebrovascular disease (Table 1).

**Table 1:** Characteristics of patients in intermediate-high intermediate-low group.

Variables	Intermediate-high (*n* = 78)	Intermediate-low (*n* = 50)	*P* value
Age, years	67.6 ± 7.3	65.2 ± 11.0	0.191
Male, *n* (%)	32 (41.0)	22 (44.0)	0.740
Smoking, *n* (%)	20 (25.6)	12 (24.0)	0.834
Hypertension, *n* (%)	48 (61.5)	32 (64.0)	0.779
Diabetes mellitus, *n* (%)	6 (7.7)	6 (12.0)	0.536
Cerebrovascular disease, *n* (%)	12 (15.4)	6 (12.0)	0.591
Chest Pain, *n* (%)	8 (10.3)	6 (12.0)	0.758
Syncope, *n* (%)	20 (25.6)	12 (24.0)	0.834
Dyspnea, *n* (%)	74 (94.9)	46 (92.0)	0.711
SBP, mmHg	132.7 ± 19.5	133.6 ± 19.9	0.796
DBP, mmHg	87.8 ± 12.5	86.9 ± 13.2	0.706
HR, bpm	95.5 ± 14.3	84.2 ± 13.2	<0.001
Respiratory rate, bpm	21.2 ± 2.5	20.4 ± 3.3	0.092
Recent operation/trauma, *n* (%)	10 (12.8)	8 (16.0)	0.614
Tumor History, *n* (%)	2 (2.6)	2 (4.0)	0.643
D-Dimer, mg/L	5.3 (2.7,9.3)	5.6 (3.4,8.3)	0.578
CTnI, mmol/L	0.14 (0.06,0.38)	0.08 (0.05,0.22)	0.087
NT-proBNP, pg/ml	1,954.0 (1313.0,4070.0)	810.0 (368.8,1430.0)	<0.001
RVD, *n* (%)	78 (100)	16 (32)	<0.001
DVT, *n* (%)	60 (76.9)	38 (76)	0.904
Lactate, mmol/L	1.1 (0.9,1.4)	1.0 (0.8,1.2)	0.107
Days of hospitalization, day	6 (5,8)	5 (5,7.3)	0.220
sPESI	2.0 (1.0,3.0)	1.0 (1.0,2.0)	<0.001
sST2, ng/ml	25.8 (12.2,50.0)	11.5 (8.1,16.0)	<0.001
Adverse event, *n* (%)	22 (28.2)	4 (8.0)	0.006

SBP, systolic blood pressure; DBP, diastolic blood pressure; HR, heart rate; CTnI, cardiac troponin I, NT-proBNP, N-terminal pro-brain natriuretic peptide; RVD, right ventricular dysfunction; DVT, deep vein thrombosis; sST2, soluble growth stimulation expressed gene 2.

Compared with the intermediate-low risk group, higher proportion of RVD (100.0% vs. 32.0%, *P* < 0.001), higher levels of sPESI (2.0, 1.0–3.0 vs. 1.0, 1.0–2.0, *P* < 0.001), NT-proBNP (1,954.0, 1,313.0–4,070.0 pg/ml vs. 810.0, 368.8–1,430.0 pg/ml, *P* < 0.001), sST2 (25.8,12.2–50.0 ng/ml vs. 11.5,8.0–16.0 ng/ml, *P* < 0.001) and more adverse events (28.2% vs. 8.0%, *P* = 0.006) were observed in the intermediate-high risk group (Table 1).

Similarly, a higher proportion of RVD (92.3% vs. 68.6%, *P* = 0.015), higher levels of sPESI (2.0, 2.0–4.0 vs. 2.0, 1.0–3.0, *P* = 0.002), NT-proBNP (4,559.0, 2,932.8–8,482.0 pg/ml vs. 1,234.0, 638.0–3,205.0 pg/ml, *P* < 0.001) and sST2 (44.9, 24.1–72.8 ng/ml vs. 13.3, 8.8–26.0, *P* < 0.001) were observed in the adverse event group compared to the no adverse event group. In addition, higher levels of CTnI (0.21, 0.12–0.44 mmol/L vs. 0.09, 0.05–0.30, *P* = 0.011) and lactate (1.3, 1.0–1.5 mmol/L vs. 1.0, 0.8–1.3, *P* = 0.004) were also significantly higher in the adverse event group compared to the no adverse event group (Supplementary Table S1).

### The association between sST2 and intermediate-high risk

Univariate logistic regression analysis showed that sST2 was associated with intermediate-high risk stratification (OR = 1.085, 95%CI 1.042–1.129, *P* < 0.001). Age, gender, HR, CTnI, NT-proBNP, adverse event and sST2 were included to construct a multifactorial regression equation, and it was found that sST2 was still associated with intermediate-high risk stratification (OR = 1.071, 95%CI 1.018–1.127, *P* = 0.008) (Supplementary Table S2).

**Figure 1:**
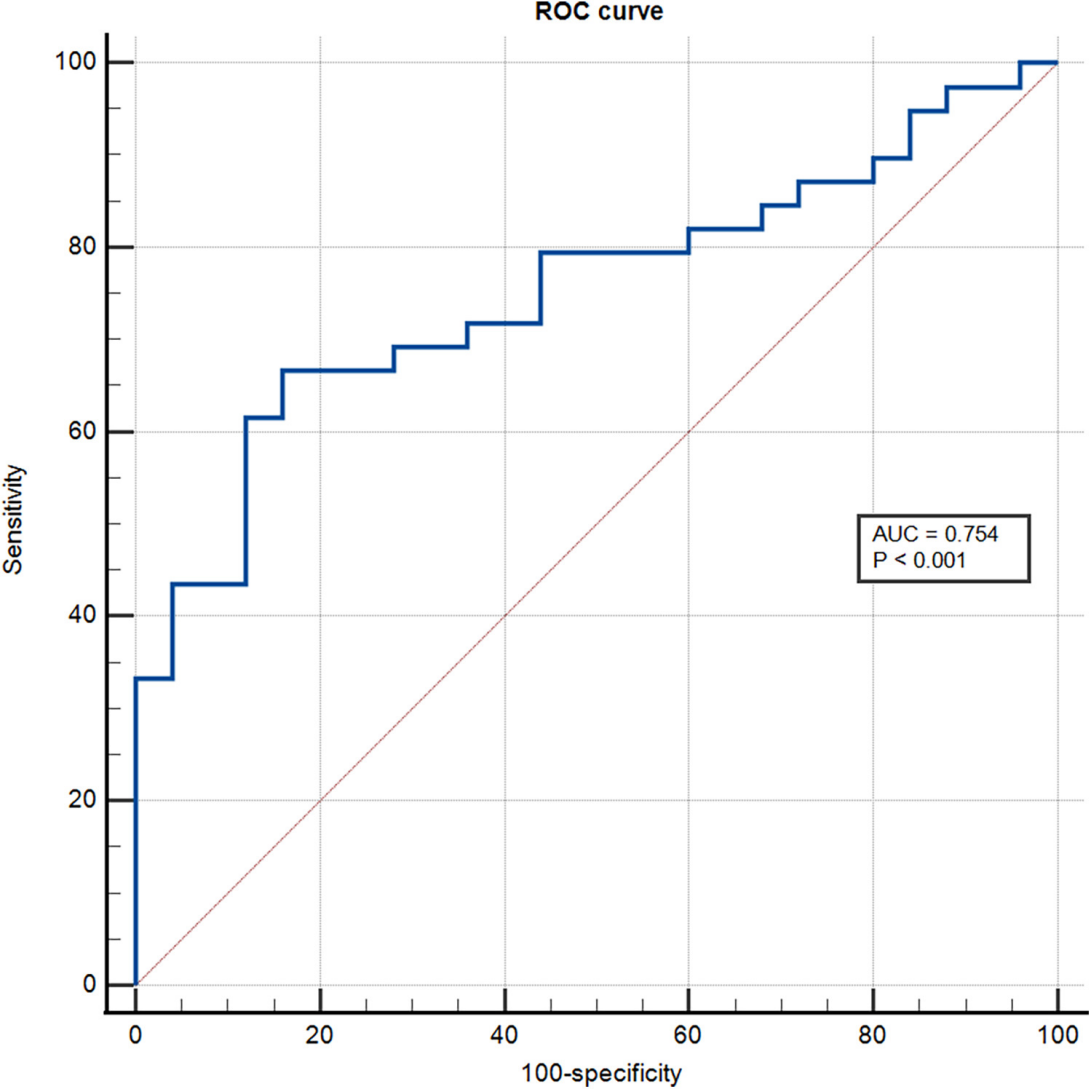
ROC curve for predicting intermediate-high risk stratification.

The ROC analysis showed that the area under the curve for sST2 identification of the intermediate-high risk stratification was 0.754 (95% CI: 0.671–0.837; *P* < 0.001), and the optimal cut-off value was 16.20 ng/ml with a sensitivity of 66.70% and specificity of 84% (Figure 1).

### The association between sST2 and adverse event

Univariate logistic regression analysis showed that sST2 was associated with adverse event (OR = 1.049, 95%CI 1.027–1.072, *P* < 0.001). After adjusting for confounding factors (age, gender, CTnI, NT-proBNP, intermediate-high risk stratification, RVD, DBP, Lac), sST2 was still associated with adverse event (OR = 1.038, 95%CI 1.007–1.070, *P* = 0.017). Other influencing factors were NT-proBNP (OR = 1.001, 95%CI 1.000–1.001, *P* = 0.002), DBP (OR = 0.910, 95%CI 0.841–0.984, *P* = 0.018) (Table 2).

**Table 2:** The multivariate logistic regression analysis of adverse event.

Variates	Adverse event					
	B	SE	Wald *χ*^2^	*P*	OR	95%CI
sST2,ng/ml	0.036	0.012	9.599	0.002	1.037	1.013–1.061
NT-proBNP, pg/ml	0.001	0.000	12.115	<0.001	1.001	1.000–1.001
DBP, mmHg	−0.085	0.033	6.599	0.010	0.919	0.861–0.980

sST2, soluble growth stimulation expressed gene 2; NT-proBNP, N-terminal pro-brain natriuretic peptide; DBP, diastolic blood pressure.

Values of sST2 and BNP in predicting adverse event.

**Figure 2:**
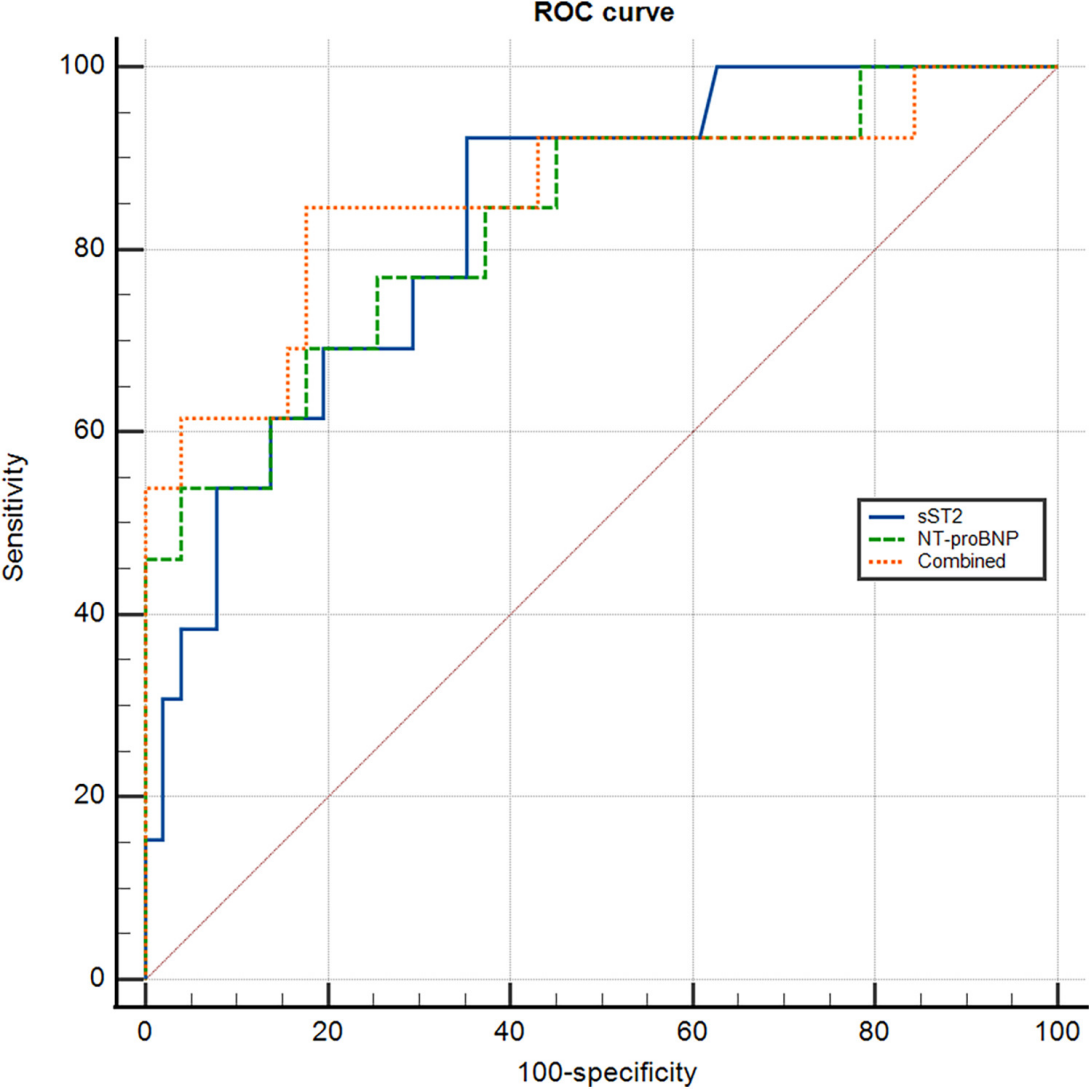
ROC curve for predicting adverse event.

The values of sST2 and NT-proBNP in predicting adverse event were analyzed by ROC curve (Figure 2).

Regarding sST2, the area under the ROC curve (AUC) for predicting adverse event was 0.832 (95% CI 0.751–0.913; *P* < 0.001). The optimal cut-off value was 16.20 ng/ml with a sensitivity of 92.30% and specificity of 64.47%. For NT-proBNP, the AUC was 0.830(95% CI: 0.735–0.924; *P* < 0.001), and the optimal cut-off value was 3581.50 pg/ml with a sensitivity of 69.20% and specificity of 82.40%. When combining sST2 and NT-proBNP, the AUC was 0.860 (95% CI: 0.765–0.954; *P* < 0.001). The optimal cut-off value was 0.173 with a diagnostic sensitivity of 84.60% and specificity 82.40% (Table 3).

**Table 3:** ROC curves for predicting adverse event.

Variates	Adverse event					
	AUC	*P*	95%CI	Cut-off value	Sensitivity	Specificity
NT-proBNP	0.830	<0.001	0.735–0.924	3,581.50 pg/ml	69.20%	82.40%
sST2	0.832	<0.001	0.751–0.913	16.20 ng/ml	92.30%	64.47%
Combined	0.860	<0.001	0.765–0.954	0.173	84.60%	82.40%

sST2, soluble growth stimulation expressed gene 2; NT-proBNP, N-terminal pro-brain natriuretic peptide.

## Discussion

The present study elicited two main findings. First, sST2 is associated with risk stratification and poor short-term prognosis for intermediate-risk APE. Second, sST2 has good predictive value for adverse events during hospitalization in intermediate-risk APE.

It is well known that risk stratification for APE is primarily based on the risk of early (hospitalization or within 30 days) death and necessary to determine the appropriate treatment. The 2019 ESC guidelines for the diagnosis and management of APE classify pulmonary embolism based on clinical presentation, laboratory, and imaging parameters. High-risk APE defined by the presence of hemodynamic instability with a high risk of death, requires urgent reperfusion therapy. Low-risk APE characterized by preserved right ventricular (RV) function, normal blood pressure and cardiac biomarkers, with a very low mortality rate. Hemodynamically stable patients who were not part of the low-risk group were considered to have an intermediate risk of mortality and complications and were defined according to PESI score or sPESI score. Included in the sPESI score are advanced age, increased heart rate, decreased blood pressure, decreased oxygen saturation, and comorbid cardiac insufficiency and tumors. The intermediate-risk group was further differentiated by imaging RVD and biomarkers (troponin and NT-ProBNP, etc.) to further distinguish intermediate-low risk from intermediate-high risk patients (5). In our study, heart rate, RVD, and NT-ProBNP levels did differ between the two subgroups, consistent with previous studies (10, 11, 12). However, for CTnI, we did not find statistical differences between risk stratification, which may be related to the inclusion of a moderate risk patient population as well as the small sample size and the overall severity of the disease.

Besides, risk stratification strategies for APE are still evolving. In intermediate-risk patients, further accurate risk stratification to identify high-risk individuals is of great importance. In a meta-analysis investigating 1680 patients with PE (13), heart-type fatty acid binding protein (H-FABP) concentrations greater than or equal to 6 ng/ml were associated with poor short-term outcomes. Another study found that patients with two cardiac biomarkers performed worse than those with one cardiac biomarker in an intermediate-high risk group (14). Therefore, the presence of superimposed cardiac biomarkers should be considered. For imaging specific metrics, right atrial enlargement (RAE)**,** the ratio between tricuspid annulus plane excursion and pulmonary arterial systolic pressure (TAPSE/PASP ratio) were found to be better predictors of prognosis independently than CTPA and CTnI in intermediate-risk APE (15). In addition, growth differentiation factor-15 (GDF-15) (16) and lactate (17), creatinine (18), hyponatremia (19), and even copeptin, a surrogate marker of antidiuretic hormone (20), have also been found to be used for risk stratification in patients with APE, and these factors need further validation. ST2 is a member of the interleukin 1 receptor family and is present in both transmembrane and soluble heterodimers. The functional ligand of ST2 is the cytokine interleukin 33 (IL-33), which is an important mediator of inflammation and immunity in several disease states (21). sST2, a soluble form of ST2, can modulate the inflammatory response and exert proinflammatory effects when secreted into the circulation. Elevated serum levels of sST2 have been observed in patients with several inflammatory and autoimmune diseases, including inflammatory bowel disease, asthma, and rheumatoid arthritis (6). In recent years, sST2 is emerging as a new biomarker in patients with cardiovascular disease. Martinez-Rumayor (7) et al. reported elevated sST2 concentrations in patients with pulmonary disease, in which case the biomarker could predict the risk of death. Pulmonary artery endothelial cellular expression of sST2, suggests that sST2 is a more pulmonary vascular specific marker for pulmonary hypertension. Patients with high sST2 levels have a worse prognosis than those with low sST2 levels in patients with pulmonary hypertension; therefore, sST2 levels can be used for risk stratification of patients with pulmonary hypertension (22). Based on this, our hypothesis that sST2 is associated with APE risk stratification. In our study, we found for the first time that elevated sST2 levels at admission in patients with intermediate-risk APE were associated with risk stratification and short-term prognosis of patients with APE, suggesting that sST2 may be a biomarker worthy of further study. Compared with the commonly used markers NT-proBNP and troponin, sST2 was not significantly correlated with multiple factors such as age, gender, renal function and body mass index. The role of sST2 in APE may be different from that of troponin and NT-proBNP, and it may be important to further investigate the factors influencing it.

A similar recently published study enrolled 72 APE patients and 38 healthy subjects [9]. The study assessed the relationship between sST2 and PESI scores and respiratory function, and evaluated the prognosis and severity performance of different levels of sST2. APE patients were found to have significantly higher sST2 levels compared to healthy individuals. PESI scores and serum lactate values were higher in the group of patients with sST2 > 35 ng/ml compared to those with sST2 < 35 ng/ml. Furthermore, sST2 was the strongest parameter discriminating against the occurrence of acute respiratory failure and PESI scores >106 relative to C-reactive protein (CRP), creatinine, d-dimer, and serum lactate. It is clearly shown that sST2 is significantly increased in APE and its elevation correlates with the severity of the disease. The authors suggested that sST2 could be used as a clinical marker to evaluate the severity of APE. However, this study did not further stratify patients at intermediate risk and lacked prognostic analysis.

Several studies have attempted to assess the prognostic value of elevated BNP and NT-pro-BNP levels in patients with APE with or without hemodynamic compromise. Optimal thresholds of 100 pg/ml for BNP and 600 pg/ml for NT-proBNP are considered useful in identifying patients at high risk for complications (5). In the present study, the threshold of NT-proBNP for identifying adverse events in patients with intermediate -risk APE exceeded the above studies. Intermediate-risk APE represents a more homogeneous group of individuals at moderate risk. Being in and about to develop RVD at different time points also complicates the discrimination and thus seems to be explainable. In addition, the lower-than-normal reference sST2 levels used to predict the occurrence of adverse events in this study may be related to the smaller sample size.

### Study limitations

Some limitations should be noted in this study. First, this is a retrospective analysis of a small number of patients from a single center. Although we suggested that sST2 was the independent predictor of adverse event, this was driven by only 26 adverse events. Thus, the results need to be confirmed by a large study population. Given the location and rank of our center, we call for relevant studies in other centers. Second, we only reviewed the adverse events during the patients’ hospitalization and did not follow them up, and we should have followed them for a longer period of time to predict their long-term adverse effects.

## Conclusions

sST2 is associated with risk stratification and short-term poor prognosis for intermediate-risk APE, and it is a promising new biomarker that may contribute to further stratification for intermediate-risk subgroups and identification of individuals with a propensity to develop adverse events during hospitalization. Combining other indicators to guide treatment options in the challenging population of intermediate-risk pulmonary embolism may be of great importance.

## Data Availability

The original contributions presented in the study are included in the article/Supplementary Material, further inquiries can be directed to the corresponding authors.

## Supplementary material

Supplementary Table S1 Characteristics of patients with or without adverse event.

Supplementary Table S2 The multivariate logistic regression analysis of Intermediate-high risk stratification.
