# Supplementary material for: The value of sST2 in risk stratification and short-term prognosis of acute pulmonary embolism: a pilot study focusing on intermediate-risk subgroups
Source: Front Cardiovasc Med. 2025 Jul 2;12:1588996. doi: 10.3389/fcvm.2025.1588996 (PMC12263577; doi:10.3389/fcvm.2025.1588996)
Supplement: Supplementary Table S1 — Characteristics of patients with or without adverse event. [file Table1.docx]

Table S1 Characteristics of patients with or without adverse event.

| Variables | Adverse event  (n=26) | No adverse event  (n=102) | P value |
| --- | --- | --- | --- |
| Age, years | 70.1±8.1 | 65.8±9.0 | 0.029 |
| Male, n(%) | 12(46.2) | 42(41.2) | 0.646 |
| Smoking, n (%) | 8(30.8) | 24(23.5) | 0.447 |
| Hypertension, n (%) | 16(61.5) | 64(62.7) | 0.910 |
| Diabetes mellitus, n (%) | 0(0) | 12(11.8) | 0.124 |
| Cerebrovascular disease, n (%) | 8(30.8) | 10(9.8) | 0.011 |
| Chest Pain, n (%) | 6(23.1) | 8(7.8) | 0.037 |
| Syncope, n (%) | 8(30.8) | 24(23.5) | 0.447 |
| Dyspnea, n (%) | 26(100) | 94(92.2) | 0.358 |
| SBP, mmHg | 130.9±20.7 | 133.6±19.4 | 0.532 |
| DBP, mmHg | 81.5±11.0 | 89.0±12.7 | 0.007 |
| HR, bpm | 91.5±14.4 | 89.5±16.9 | 0.533 |
| Respiratory rate, bpm | 20.2±2.7 | 21.0±2.9 | 0.187 |
| Recent operation/trauma, n (%) | 2(7.7) | 16(15.7) | 0.526 |
| Tumor History, n (%) | 0(0) | 4(3.9) | 0.582 |
| D-Dimer, mg/L | 5.8(2.7,7.1) | 4.9(3.4,9.3) | 0.924 |
| CTnI, mmol/L | 0.21(0.12,0.44) | 0.09(0.05,0.30) | 0.011 |
| NT-proBNP, pg/mL | 4559.0(2932.8,8482.0) | 1234.0(638.0,3205.0) | <0.001 |
| RVD, n (%) | 24(92.3) | 70(68.6) | 0.015 |
| DVT, n (%) | 20(76.9) | 78(76.5) | 0.961 |
| Intermediate-high, n (%) | 22(84.6) | 56(54.9) | 0.006 |
| Lactate,mmol/L | 1.3(1.0,1.5) | 1.0(0.8,1.3) | 0.004 |
| Days of hospitalization, day | 6(5,7) | 6(5,8) | 0.773 |
| sPESI | 2.0(2.0,4.0) | 2.0(1.0,3.0) | 0.002 |
| sST2,ng/mL | 44.9(24.1,72.8) | 13.3(8.8,26.0) | <0.001 |

Abbreviations: SBP, systolic blood pressure; DBP, diastolic blood pressure; HR, heart rate; CTnI, cardiac troponin I, NT-proBNP, N-terminal pro-brain natriuretic peptide; RVD, right ventricular dysfunction; DVT, deep vein thrombosis; sST2, soluble growth stimulation expressed gene 2.
