# Supplementary material for: The value of sST2 in risk stratification and short-term prognosis of acute pulmonary embolism: a pilot study focusing on intermediate-risk subgroups
Source: Front Cardiovasc Med. 2025 Jul 2;12:1588996. doi: 10.3389/fcvm.2025.1588996 (PMC12263577; doi:10.3389/fcvm.2025.1588996)
Supplement: Supplementary Table S2 — The multivariate logistic regression analysis of Intermediate-high risk stratification. [file Table2.docx]

Table S2 The multivariate logistic regression analysis of Intermediate-high risk stratification.

| Variates | Intermediate-high | | | | | |
| --- | --- | --- | --- | --- | --- | --- |
|  | B | SE | Wald χ^2^ | P | OR | 95%CI |
| sST2, ng/mL | 0.061 | 0.023 | 7.216 | 0.007 | 1.063 | 1.017~1.111 |
| NT-proBNP, pg/mL | 0.000 | 0.000 | 6.422 | 0.011 | 1.000 | 1.000~1.001 |
| HR, bpm | 0.063 | 0.019 | 10.713 | 0.001 | 1.065 | 1.026~1.107 |

Abbreviations: sST2, soluble growth stimulation expressed gene 2; NT-proBNP, N-terminal pro-brain natriuretic peptide.
